# Genetic markers for urine haptoglobin is associated with decline in renal function in type 2 diabetes in East Asians

**DOI:** 10.1038/s41598-018-23407-1

**Published:** 2018-03-23

**Authors:** Resham Lal Gurung, Rajkumar Dorajoo, Sylvia Liu, Yiamunaa M, Jian-Jun Liu, Ling Wang, Lin Guo, Xueqing Yu, Jian-Jun Liu, Su Chi Lim

**Affiliations:** 1 Clinical Research Unit, Khoo Teck Puat Hospital, Singapore, Singapore; 2 Human Genetics, Genome Institute of Singapore, Agency for Science, Technology and Research, Singapore, Singapore; 3 The First Affiliated Hospital, Sun Yat-sen University, Guangzhou, China; 4 Guangdong Medical University, Guangzhou, China; 5 Yong Loo Lin School of Medicine, National University of Singapore, Singapore, Singapore; 6 Diabetes Centre, Khoo Teck Puat Hospital, Singapore, Singapore; 7 Saw Swee Hock School of Public Health, National University Hospital, Singapore, Singapore

## Abstract

Urine haptoglobin (uHP) level prospectively predicts diabetic kidney disease (DKD) progression. Here, we aim to identify genetic determinants of uHP level and evaluate association with renal function in East Asians (EA) with type 2 diabetes (T2D). Genome-wide association study (GWAS) among 805 [236 Chinese (discovery) and 569 (57 Malay and 512 Chinese) (validation)] found that rs75444904/kgp16506790 variant was robustly associated with uHP level (MetaP = 1.21 × 10^−60^). rs75444904 correlates well with plasma HP protein levels and multimerization in EA but was not in perfect LD (r^2^ = 0.911 in Chinese, r^2^ = 0.536 in Malay) and is monomorphic in Europeans (1000 G data). Conditional probability analysis indicated weakening of effects but residual significant associations between rs75444904 and uHP when adjusted on HP structural variant (MetaP = 8.22 × 10^−7^). The rs75444904 variant was associated with DKD progression (OR = 1.77, P = 0.014) independent of traditional risk factors. In an additional validation-cohort of EA (410 end-stage renal disease (ESRD) cases and 1308 controls), rs75444904 was associated with ESRD (OR = 1.22, P = 0.036). Furthermore, increased risk of DKD progression (OR = 2.09, P = 0.007) with elevated uHP level through Mendelian randomisation analysis provide support for potential causal role of uHP in DKD progression in EA. However, further replication of our findings in larger study populations is warranted.

## Introduction

Diabetes kidney disease (DKD) is the leading cause of chronic kidney disease (CKD) and end-stage renal disease (ESRD) globally. More than 50% of type 2 diabetes (T2D) patients in Asia have renal complications compared to 40% in Caucasians^1^. In Singapore, the prevalence of DKD is more than 50% in T2D patients and varies within subpopulation^2^. This is further complicated by the wide spectrum of renal progression in DKD patients, from very fast to moderate decline^3^. Therefore, this necessitates the need for prospective studies to identify biomarkers that allows stratification of DKD patients at high risk for rapid decline in renal function.

Haptoglobin (HP), an abundant serum protein, facilitates the removal of free haemoglobin (Hb) from circulation via macrophage-specific receptor CD163, preventing oxidative damage in tissues^4–7^. In observational studies, urine haptoglobin (uHP) has been associated with DKD, independent of other classical risk factors. For example, a study among Veterans Affairs Diabetes Trial (VADT) subjects demonstrated that increased uHP level is associated with early renal function decline in DKD patients^8^. In Asian cohorts, we confirmed that uHP predicted rapid DKD progression as good as, if not better than, albuminuria in T2D patients with preserved renal function (estimated glomerular filtration rate (eGFR) > 60 mL/min/1.73 m^2^)^9^. In a recent study in Chinese cohort with median follow-up of 5.3 years, Yang *et al*., showed that T2D individuals with microalbuminuria and elevated uHP level are most susceptible to development of CKD, as defined by eGFR < 60 mL/min/1.73 m^2^ ^10^. However, whether this association of uHP and renal function is causal remains unclear. For example, unmeasured lifestyle factors such as smoking habits and physical activities might confound observational studies^11,12^. Furthermore, reverse causality could similarly lead to a statistically robust but non-causal relationship^13^.

Mendelian randomisation (MR) approach using genetic markers as instrumental variable (IV), allows for the assessment of causal relationship^14^. MR is analogous to a randomized clinical trial as genetic variants are randomly assorted at conception, precede any disease and less likely to be affected by confounding or reverse causality. The human *HP* (haptoglobin) gene has two common alleles, HP1 and HP2, which differs by the absence (HP1) or presence (HP2) of a 1.7 kbp intragenic duplication resulting in three potential HP genotypes (HP 1-1, HP 2-1 and HP 2-2) and corresponding proteins that are functionally distinct^5,15,16^. To our knowledge, the genetic determinant of uHP has not been explored although series of studies have shown that HP 2-2 genotype and rs2000999 in the *HP* gene were associated with serum haptoglobin levels^17–21^. However, the association between HP polymorphism and DKD remains conflicting among different ethnic populations^22–27^. Given that urine is an important potential source of kidney biomarker^28^, we aimed to first identify genetic determinant of uHP level and subsequently examine its association with decline in renal function to assess causality between uHP and DKD in East Asians.

## Results

### Baseline characteristic of the study participants

Table 1 summarises the baseline characteristics of participants, subjected to GWAS and HP structural variants (HP1/HP2) genotyping stratified by ethnicity. Considerable differences in median or mean values for BMI, HDL-C, eGFR and uACR were observed between ethnic groups. Malays had a higher proportion of female and DKD progressors (rapid decline in eGFR) as compared to Chinese. However, the distribution of HP genotype, median uHP and plasma haptoglobin (pHP) was comparable between Chinese and Malay.

**Table 1:** Baseline clinical and biochemical characteristic of subjects stratified by ethnicity.

	Total (n = 327)	Chinese (n = 250)	Malay (n = 77)	P
uHaptoglobin (ng/ml)	203 (14–1828)	157 (20–1442)	365 (10–2790)	0.229
pHaptoglobin (µg/ml)	1077 (766–1511)	1074 (732–1487)	1132 (893–1600)	0.131
Entry Age (years)	56.9 ± 12.2	57.4 ± 12.5	55.2 ± 11.1	0.173
Gender (Female, %)	150(45.9)	104 (41.6)	46 (59.7)	**0**.**005**
BMI (Kg/m^2^)	26.8 ± 4.9	26.1 ± 4.3	28.8 ± 6.0	**<0**.**001**
HbA1c (mmol/mol)	66.3 ± 21.1	64.9 ± 19.5	70.8 ± 25.3	0.065
Diabetes Duration (years)	10.6 ± 8.0	10.6 ± 8.0	10.5 ± 8.1	0.925
Lipids profile				
TC (mM)	4.70 ± 1.02	4.69 ± 0.96	4.75 ± 1.18	0.663
HDL-C (mM)	1.27 ± 0.37	1.30 ± 0.37	1.18 ± 0.34	**0.016**
LDL-C (mM)	2.83 ± 0.77	2.82 ± 0.73	2.89 ± 0.90	0.483
Triglycerides (mM)	1.48 (1.08–2.08)	1.48 (1.07–2.09)	1.47 (1.17–2.08)	0.586
Blood pressure (mmHg)				
SBP	135 ± 20	135 ± 20	134 ± 20	0.620
DBP	78 ± 11	78 ± 10	77 ± 12	0.718
Renal function (baseline)				
eGFR (mL/min/1.73 m^2^)	80.6 ± 30.9	82.5 ± 29.9	74.5 ± 33.5	**0.048**
uACR (µg/mg)	49 (16–150)	38 (13–131)	107 (29–262)	<**0.001**
Trajectory of eGFR				
Duration of follow-up (year)	5.5 (5.0–6.7)	5.7 (5.0–7.0)	5.2 (4.3–6.0)	**0.001**
ml/min per 1.73 m^2^/year	−1.0 (−6.2 to 0.5)	−0.9 (−4.9 to 0.6)	−3.3 (−7.7 to 0.2)	**0.015**
percentage change/year	−1.3 (−8.0 to 0.6)	−1.1 (−6.4 to 0.8)	−4.3 (−11.2 to 0.2)	**0.012**
No of progressors (n, %)	123 (37.6)	83 (33.2)	40 (51.9)	**0.003**
Use of Medication (n, %)				
Insulin	113 (34.6)	80 (32.0)	33 (42.9)	0.080
RAS antagonist	226 (69.1)	166 (66.4)	60 (77.9)	0.056
HP genotype				0.447
HP 1–1	35 (10.7)	28 (11.2)	7 (9.1)	
HP 2–1	130 (39.8)	103 (41.2)	27 (35.1)	
HP 2–2	162 (49.5)	119 (47.6)	43 (55.8)	

**Abbreviations:** uHP, urine haptoglobin; pHP, plasma haptoglobin; BMI, body mass index; HbA1c, glycated haemoglobin; HDL-C, HDL cholesterol; LDL-C, LDL cholesterol; uACR, urine albumin creatinine ratio; eGFR, estimated glomerular filtration rate; RAS, renin-angiotensin system. Data are described as mean +/- SD or median (interquartile range) for skewed variables or proportion of participants (%) where appropriate. Bold values represent statistically significant data between ethnic groups.

### Genetic determinant of haptoglobin levels

**Figure 1:**
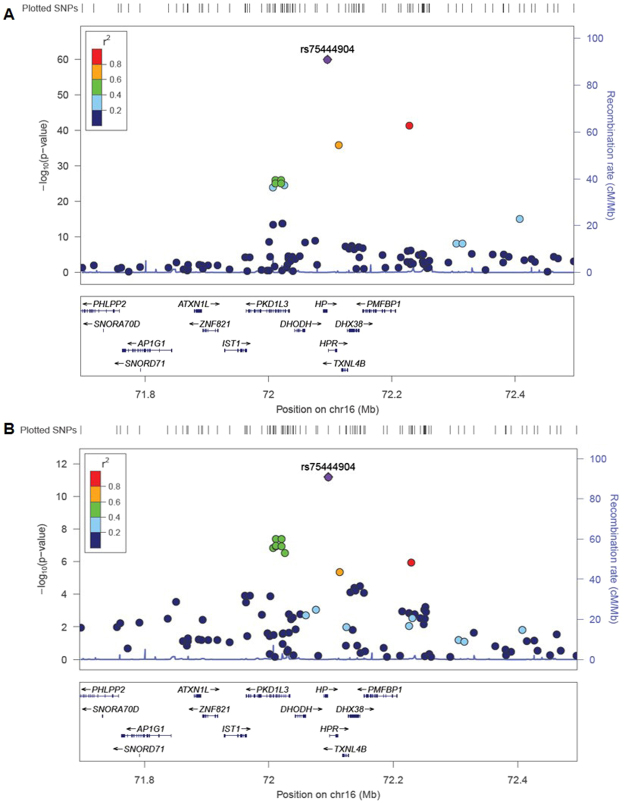
Chromosomal plot of genome-wide signal at chromosome 16 for A) urine z-HP associations and B) plasma z-HP associations. Association p-values derived from meta-analysis of discovery stage and validation stage datasets. LD (r^2^) data of the SNPs are based on ASN panels of 1000 Genome Project database. Plots generated using LocusZoom (http://locuszoom.sph.umich.edu/).

QQ-plots for urine and plasma z-HP levels in the discovery (DN Chinese) and validation (DN Malay and SMART2D Chinese) datasets are shown in Supplementary Figs S3 and S4. The genomic inflation factor (λ) at the discovery and validation stages of the study was observed to be minimal (λ between 0.990–1.027), indicating that quality control (QC) procedures had effectively excluded aberrant samples and SNPs, and analysis methods had effectively controlled for possible population stratification issues. A strong genotyped signal beyond the genome-wide significance threshold was observed at chromosome 16, corresponding to the *HP* gene locus for urine z-HP in the discovery stage (P = 1.07 × 10^−16^, Table 2). The top SNP for urine z-HP association, kgp16506790 (rs75444904) was highly polymorphic in EA populations but monomorphic in most other reference populations (1000 Genomes reference) and explained approximately 25.5% of phenotypic variance of uHP levels in our Chinese samples. The association of rs75444904 with urine z-HP levels was replicated in the Malay dataset (P = 1.80 × 10^−3^) and independent Chinese samples from SMART2D cohort (P = 3.98 × 10^−41^) (Fig. 1 and Table 2). Although rs75444904 was imputed in the SMART2D cohort, the SNP was observed with good imputation confidence score (info score = 0.86) and had comparable MAF (0.275). Repeating uHP association analyses at rs75444904 in discovery and validation stages using the mixed model association to further control for population substructure (GEMMA) or through the EIGENSTART method indicated similarly robust genome-wide associations (Supplementary Table S3). This same SNP also reached genome-wide significance level for plasma z-HP in the discovery Chinese samples (P = 2.08 × 10^−10^, Table 2), explaining approximately 14.7% of phenotypic variance of plasma HP levels in our Chinese samples. The identified rs75444904 associations with plasma z-HP levels also showed a similar trend in the Malay validation samples although this was not statistically significant (P = 0.087, Table 2).

**Table 2:** SNP with genome-wide levels of associations for urine z-HP levels in the discovery stage of the study and their corresponding association levels in the validation datasets.

SNP	Chr	Pos	TA	Discovery stage			Replication stage						Meta-analysis (N = 805)			
				DN Chinese (N = 236)			DN Malay (N = 57)			SMART2D Chinese (N = 512)			β (SE)	P	P_het	I^2^
				TAF	β (SE)	P	TAF	β (SE)	P	TAF	β (SE)	P				
*urine z-HP*																
rs75444904	16	72095650	C	0.307	0.732 (0.082)	1.07 × 10^−16^	0.219	0.705 (0.214)	1.80 × 10^−3^	0.275	0.807 (0.059)	3.98 × 10^−41^	0.777 (0.047)	1.21 × 10^−60^	0.717	0
*Plasma z-HP*																
rs75444904	16	72095650	C	0.307	0.583 (0.088)	2.08 × 10^−10^	0.219	0.438 (0.251)	0.087	NA	NA	NA	0.567 (0.083)	6.51 × 10^−12^	0.586	0

Data analysis was adjusted for age, sex and Principal Components (PC1 for Chinese and PC1–3 for Malay). **Abbreviations**: Chr, chromosome number; TA, test allele; TAF, test allele frequency, P_het: Cochran’s Q heterogeneity p-value, I^2^: heterogeneity index, NA: not available.

In a pooled analysis of participants with both pHP and uHP measured, individuals with rs75444904 CC genotype had almost 10-fold increase in uHP compared to AA genotype (Supplementary Table S4). uHP levels were significantly higher in individuals with HP 1-1 genotype and HP 2-1 genotype as compared to HP 2-2 genotype, and HP 1-1 genotype compared to HP 2-1 genotype (HP 1-1 = 4292ng/ml, HP 2-1 = 511ng/ml, HP 2-2 = 41ng/ml, P < 0.0001) (Supplementary Table S4). In contrast, pHP levels were only significantly higher in individuals with HP 1-1 and HP 2-1 genotype when compared to HP 2-2 but not in HP 1-1 genotype as compared to HP 2-1 (Supplementary Table S4). In a sensitivity analysis, excluding patients with macroalbuminuria (uACR ≥ 300 µg/mg) and eGFR less than 60 mL/min/1.73 m^2^ at baseline did not materially change the significantly elevated level of uHP level in individuals with HP1 allele compared to HP2 allele (P < 0.0001, n = 244).

### Linkage disequilibrium (LD) of rs75444904 and HP common polymorphism

HP structural variant (HP1 allele) and rs75444904 [C] allele were observed to be in LD in both the Chinese (r^2^ = 0.911) and Malay datasets (r^2^ = 0.536). Conditional probability analysis on rs75444904 and HP1 allele showed that individual associations with uHP were not completely abolished (HP 1; β = 0.264, P = 0.010 and rs75444904; β = 0.543, P = 8.22 × 10^−7^, Supplementary Table S5). Previous studies in European population suggested rs217181 as the best proxy for tagging HP allele (r^2^ = 0.44)^29^. Therefore, we looked at the association of rs217181 with haptoglobin levels in our study cohort. In combined analysis, the association of rs217181 with plasma (β = 0.384, P = 4.24 × 10^−6^) and urine (β = 0.621, P = 1.45 × 10^−36^) haptoglobin levels reached genome wide level but was weaker compared to rs75444094 (Supplementary Table S6). Moreover, conditioning on rs75444904 genotypes attenuated the genome wide association of rs217181 and urine HP (P = 0.003) while the association remained robustly significant at rs75444904 (P = 8.64 × 10^−23^) (Supplementary Table S7).

### Association of genotype and renal functions

After meta-analysis, rs75444904 SNP was significantly associated with a 77% increased risk for DKD progression after adjustment for age, sex, principle components, BMI, HbA1c, diabetes duration, systolic blood pressure, HDL-C, LDL-C, TG, eGFR, uACR and intake of insulin and RAS antagonist (OR = 1.77, 95% CI 1.32–2.23, p = 0.014) (Table 3, Supplementary Table S8). We also observed significant association of HP1 allele with increased risk for DKD progression (OR = 1.91, 95% CI 1.45–2.37, p = 0.006) (Table 3, Supplementary Table S8).

**Table 3:** Association between rs75444904 and HP1 allele with DKD progression under additive model in East Asians.

	Progressors	Non-Progressor	Meta-analysis		
			OR (95% CI)	P	P_het
rs75444904 (AA/CA/CC)	45/53/10	107/59/19	1.77 (1.32–2.23)	**0.014**	0.425
HP1 (1–1/2–1/2–2)	15/59/49	20/71/113	1.91 (1.45–2.37)	**0.006**	0.084

Data represents odd ratios (OR) and 95% CI adjusted for age, gender, HbA1c, diabetes duration, Systolic BP, lipids (natural log-transformed HDL-C, LDL-C and TG), eGFR, uACR (natural log-transformed) and RAS antagonist and insulin usage. **Abbreviations**: P_het: Cochran’s Q heterogeneity p-value. Bold values represent statistically significant data.

We further examined the association of rs75444904 with ESRD cross-sectionally in independent samples from our Diabetic Nephropathy (DN), SMART2D cohorts^30^ and T2D samples from Chinese cohort from China. After meta-analysis, we observed modest but significant association of rs75444904 with 22% increased risk for ESRD (410 cases vs 1308 control; OR = 1.22, 95% CI 1.01–1.47, P = 0.036) after adjusting for age, gender and principle components (except in the China dataset where GWAS data was not available) (Table 4).

**Table 4:** Association of rs75444904 with ESRD in independent samples in East Asians.

	DN		SMART2D		CH	Meta-Analysis
	Chinese	Malay	Chinese	Malay	Chinese	
*N*	881	224	391	105	117	1718
rs75444904 (AA/CA/CC)	441/346/94	132/78/14	210/153/28	59/30/16	59/42/16	901/649/168
MAF (C allele)	0.302	0.237	0.267	0.295	0.316	0.287
ESRD (Case/Control)	194/687	102/122	18/373	14/91	82/35	410/1308
OR	1.132	1.402	1.354	1.214	1.213	1.22
P	0.304	0.134	0.412	0.662	0.523	0.036
95% CI						1.01–1.47
P_het						0.898

**Abbreviations:** MAF, minor allele frequency; P_het, Cochran’s Q heterogeneity p-value. ESRD case is defined as eGFR < 15 mL/min/1.73 m^2^ and control is defined as T2D patients with diabetes duration of more than 10 years and eGFR > 15 mL/min/1.73 m^2^. Data not adjusted for PCs in CH dataset without GWAS data.

### Mendelian Randomisation analysis

Given the robust association with uHP level and significant association with renal decline, we used rs75444904 as an instrumental variable for uHP for MR analysis. We found a significant association between genetically increased uHP and risk for DKD progression in our study. After meta-analysis of all datasets, 1 SD increase in uHP was associated with DKD progression (OR = 2.09 95% CI 1.50–2.67, P = 0.007) (Table 5) after adjustment for DKD traditional risk factors. Given the LD between rs75444904, HP structural variant and rs217181, we found similarly significant observations using HP1 or rs217181 as instrumental variable (Table 5).

**Table 5:** Mendelian Randomisation analysis on association of uHP with DKD progression in T2D patients.

Instrumental variable	Linkage disequilibrium (r2) with HP 1 (Chinese/Malay)	Effect/Other allele	EAF (%) (Chinese/Malay)	beta (SE) of urine Haptoglobin level per allele	P	OR (95% CI) for DKD progression per allele	P	MR estimate of DKD progression per 1 SD increase in uHP	P
HP 1	—	HP1/HP2	0.291/0.267	0.727 (0.046)	1.42 × 10^−55^	1.91 (1.45–2.37)	0.006	2.42 (1.80–3.06)	0.003
rs75444904	0.91/0.54	C/A	0.285/0.219	0.777 (0.047)	1.21 × 10^−60^	1.77 (1.32–2.27)	0.014	2.09 (1.50–2.67)	0.007
rs217181	0.84/0.45	T/C	0.324/0.272	0.621 (0.048)	1.45 × 10^−36^	1.83 (1.37–2.29)	0.010	2.64 (1.90–3.38)	0.005

Data were adjusted for age, gender, HbA1c, diabetes duration, Systolic BP, lipids (natural log-transformed HDL-C, LDL-C and TG), eGFR, uACR (natural log-transformed) and usage of medications.

HP1 allele has been associated with reduced level of LDL cholesterol^29^. Mediation analysis^31^ for DKD progression in our study, adjusted for traditional risk factors, suggested full mediation by uHP, and in contrast, lack for mediation by LDL-C (Supplementary Table S10).

## Discussion

In this study, we identified a robust GWAS signal rs75444904 (upstream of *HP* gene) as an East Asian specific variant influencing uHP level. Genetic disposition to higher uHP level in T2D patients was associated with higher risk for DKD progression independent of traditional risk factors including hypertension, hyperglycemia, diabetes duration, dyslipidemia as well as baseline renal function and usages of medications. Individuals with rs75444904 risk variant allele were also at 22% increased risk for progression to ESRD. We further used a MR approach to provide genetic evidence to support the potential causal relationship of increased uHP level in DKD progression in East Asians. These are novel observations as no other studies have reported genetic markers for uHP and used MR approach to assess the casual association in East Asian populations.

In the current analysis, elevated uHP levels were observed in T2D individuals with rs75444904 [C] allele and HP1 allele as compared to rs75444904 [A] and HP2 allele respectively. Interestingly, rs75444904 is monomorphic in Europeans (1000 Genome database) but has minor allele frequency between 20–30% in the Chinese and Malays. Conditional probability analysis did not completely abolish the individual associations of rs75444904 [C] allele and HP1 allele with uHP. Moreover, the association of rs75444904 with uHP was stronger compared to HP 1 allele (HP 1; β = 0.264, P = 0.010 and rs75444904; β = 0.543, P = 8.22 × 10^−7^). Therefore, we cannot rule out that rs75444904 SNP may have some independent effect or may also tag to another causal mutation. A recent large scale study aimed to identify tagging SNPs for HP structural variant in Europeans found that HP allele is correlated with rs217181 (r^2^ = 0.44)^29^. In East Asians, we found that rs75444904 and HP allele is highly correlated (Chinese, r^2^ = 0.911; Malay, r^2^ = 0.536). More importantly, we observed that the association of rs217181 with uHP level is weaker compared to rs75444904 and driven mainly by rs75444904 at this locus. This suggests that rs75444904 may be a better surrogate as genetic marker for uHP level in T2D in East Asians.

Findings from this study provides genetic support to previous observational studies by us and others demonstrating association of uHP with decline in renal function in T2D patients^8–10^. For MR analysis, a strong link between the genetic variant used as the instrumental variable and the exposure (uHP level), as demonstrated in our study, is essential. Recent reports have shown that HP structural variant is associated with reduced LDL cholesterol levels^29^. However, the likelihood of horizontal pleiotropy is minimal in our study for 1) the SNP or copy number variant explains a significantly high proportion of phenotypic variability (20–30% of uHP variance); 2) the uHP is instrumented in MR by cis-acting variant in the vicinity of the encoding gene^32^; 3) the association of genetic instrument with the disease outcome (DKD progression) is mediated solely through the biomarker of interest (urine haptoglobin) and not LDL-C and 4) associations between the genetic instrumental variable and DKD progression remained significant after adjustments for traditional risk factors and measures of population structure (principle components). Nevertheless, it remains possible that pleotropic effects of the genetic instrument variable used in our study may still exist with other unknown and unmeasured factors and these may confound study results.

Most of the studies on evaluating the biological role of HP1 allele are in relation to its enhanced anti-oxidative function as compared to HP2 allele in cardiovascular disease^33^. Haptoglobin is also an angiogenic factor and is essential for functional role of endothelial cells in neovascular development^34^. Besides the liver, *HP* gene is also expressed in the kidney and inducible through cytokines such as interleukin-6^35^. From our study, uHP level among HP2 individuals does not differ between subjects with impaired or preserved renal function, suggesting that increase in uHP level may probably be due to increase *in-situ* expression in renal tissues. Data from mouse models subjected to acute kidney injury with multiple agents revealed relatively greater and sustained increase in renal (proximal tubules) expression of *HP* as compared to hepatic *HP* expression^36^. While the focus of this study was to assess the causal relationship between uHP and renal function, undeniably, further mechanistic data, as well as evidence from prospective studies, are required to confirm the role of the rs75444904 in the pathophysiology of renal complications of diabetes. In line with this, recent studies have highlighted HP1 allele as a risk factor for white matter hyperintensities and stroke^37,38^. Compared to uHP, the genetic determinants of serum haptoglobin level has been previously reported with rs2000999, rs5472 variants in *HP* gene and HP structural variants affecting the serum haptoglobin level^17,19,20^. In our study, we also found that rs75444904 and HP1 allele are robustly associated with pHP level although the association was weaker as compared to that with uHP level. However, pHP level was not associated with DKD progression in T2D patients after adjusting for traditional risk factors. Serum haptoglobin level are modulated by various clinical disease such as inflammation, haemolysis and liver disease^39,40^ and thus may less likely be an ideal reflection of patients’ renal conditions. Therefore, our findings demonstrate the added advantages of using urine samples for identifying novel biomarker in pathologies of DKD.

Our study represents the first comprehensive search for genetic determinant of uHP levels using GWAS in East Asians with T2D. Additionally we compared both pHP and uHP level in the same individual to demonstrate the utility of uHP as a better predictor and causal factor for DKD progression. Moreover, DKD progression was defined by trajectory slope ensuring gradual decrease in T2D patients with a median follow-up of 5.5 years and more than 3 eGFR readings. However, we acknowledge limitations of our study. To efficiently analyses DKD progression biomarkers, we only included T2D patients with at least 3 eGFR readings to generate a trajectory slope to classify them as rapid progressors and non-progressors which resulted in a relatively small sample size for GWAS (n = 327). Despite the small sample size, robust signal was observed for association of common variants and uHP level. Our success in part could be due to 1) “enriched” phenotype study design used in the initial GWAS which may have increased the power of our study; 2) genetic variant identified (rs75444904) is in close proximity or within the gene coding the protein (HP copy number variant), suggesting fewer biological steps between genetic variant and protein synthesis and a larger signal to noise ratio^41^; 3) use of intermediate phenotype eGFR gradient as outcome; 4) relatively higher frequency of minor allele (MAF~0.33) among East Asians. While we validated the association of rs75444904 with ESRD cross-sectionally in larger independent East Asian samples, further large scale studies in independent East Asian population with long term follow-up data would be necessary to firmly confirm or dismiss the association at this locus.

In conclusion, we have identified East Asian specific common variant rs75444904 that influences uHP level and demonstrated that a genetic predisposition to increase uHP level was associated with increased risk of decline in renal function in T2D. These findings provide evidence supportive of a potential causal link between uHP and renal function in T2D. If further validated in independent cohorts, therapy targeting uHP may likely be effective in delaying DKD progression in East Asians.

## Materials and Methods

### Study design and participants

Participants for this study were recruited in the diabetes centres in Singapore as described previously in our Diabetic Nephropathy (DN) cohort^9^. Briefly, the exclusion criteria were as follows: 1) age below 21 years; 2) pregnancy; 3) manifest infectious disease, active cancer, and autoimmune diseases; or 4) type 1 diabetes (requirement for continuous insulin therapy within 1 year after diabetes onset or acute presentation with ketoacidosis). 327 participants (250 Chinese and 77 Malays) from the DN study were subjected to genome wide association study. Top index SNP was further validated in 512 independent Chinese samples from previously reported SMART2D cohort^30^.

### Definition of Outcomes

Primary renal outcome in the prospective analysis was DKD progression as described previously in our DN study using the same cohort^9^. DKD progression was defined as decline in eGFR gradient > 3 mL/min/1.73 m^2^ per year by trajectory slope^3^. Non-progressors were  defined as eGFR changes ± 2 mL/min/1.73 m^2^ per year and at least 5 years follow-up. In total, 327 T2D participants comprised of 83 progressors and 167 non-progressors in Chinese subjects and 40 progressors and 37 non-progressors in Malay subjects.

For secondary cross-sectional analysis on association of our candidate SNP with renal disease traits, ESRD patients at baseline were defined as T2D patients with eGFR < 15 mL/min per 1.73 m^2^ while the controls for ESRD were T2D patients with diabetes duration for more than 10 years with eGFR > 15 mL/min per 1.73 m^2^. The information on the DN and SMART2D cohorts used for secondary analysis has been detailed previously^30^. The Chinese samples consist of 82 T2D-related ESRD cases and 34 T2D-related controls, collected during the period from October 2015 to December 2016 from 7 different hospitals in China (Supplementary Methods). Written informed consent was obtained from all participants. This study complies with Helsinki Declaration and has been approved by the Singapore National Health Group (NHG) domain specific ethical review board.

### Clinical and biochemical measurement

Total urine and plasma haptoglobin was measured using a sandwich enzyme immunoassay (R&D Systems Inc.) according to the manufacturer’s instruction. Urinary albumin: creatinine ratio (ACR) was determined by urinary creatinine measured by the enzymatic method on a cobas c system (Roche Diagnostics, Mannheim, Germany) and albumin measured by a solid-phase competitive chemiluminescent enzymatic immunoassay with a lower detection limit of 2.5 lg/mL (Immulite, DPC, Gwynedd,UK). The baseline and follow- up eGFR was calculated based on the widely used Modified Diet in Renal Disease equation in patients with diabetes.

### Genotyping

#### Haptoglobin structural (copy number) variant

PCR followed by gel electrophoresis was used to genotype for the HP structural variant (HP1/HP2) in 327 subjects from DN cohort as reported previously^42^. This procedure produces unique PCR products for each HP alleles. We performed duplicates for selected samples to demonstrate 100% concordance.

#### Genome-wide association study

Genome-wide genotyping was performed for 2,664 samples from the DN cohort using the Illumina HumanOmniZhonghua Bead Chip. Quality control (QC) procedures of samples and SNPs are detailed in Supplementary Table 1. Briefly, samples with call-rate < 95.0% (N = 4), extremes in heterozygozity (>3 SD from mean, N = 55) and known duplicates (N = 12) were excluded from analyses. Identity-by-state measures were performed by pair-wise comparison of samples to detect 1st degree related samples (Supplementary Table 1) and one sample from each relationship was excluded from further analysis (N = 96). Principle component analysis (PCA) together with 1000 Genomes Projects reference populations was performed to identify possible outliers from reported ethnicity and 127 samples were excluded (Supplementary Figs 1 and 2). After GWAS sample QC procedures, 236 Chinese and 57 Malays with uHP and pHP information were available for subsequent statistical analysis (Supplementary Table S1).

For SNP QC, sex-linked and mitochondrial SNPs were removed, together with gross HWE outliers (p-value < 1 × 10-4) (Supplementary Table S2). SNPs that were monomorphic or with a MAF < 1.0% and SNPs with low call-rates (<95.0%) were excluded (Supplementary Table S2). Alleles for all SNPs were coded to the forward strand and mapped to HG19.

### Statistical analysis

#### GWAS discovery stage urine and plasma Haptoglobin associations

To identify SNPs that influence urine and plasma HP levels, we first performed a discovery stage analysis using data from DN Chinese subjects. Raw urine and plasma HP levels were normalized by rank-based inverse normalization (z-scores). Linear regression analyses between SNPs and urine z-HP and plasma z-HP levels were performed using PLINK v1.9 in an additive model and adjusted for age, sex and population stratification (PC1). Genomic inflation factor (λ) of association results was used to evaluate levels of inflation of study results. SNPs with association p-values that reached genome-wide levels (p-value < 5 × 10^−8^) were followed up in the validation stage of the study. For lead SNPs with genome-wide association identified in our study, we estimated the proportion of the respective urine and plasma HP variance explained by evaluating adjusted R^2^ values of regression models before and after inclusion of the lead SNP into the model.

#### GWAS validation stage plasma and urinary Haptoglobin associations

uHP and pHP data from DN Malay subjects were utilized in the validation stage of the study. In addition, the uHP were measured in additional independent 512 Chinese samples (SMART2D cohort) with GWAS data from our previous study^30^. As in discovery samples, raw values were normalized as indicated above. Genome-wide linear regression analyses between SNPs and z-HP and urine z-HP were performed separately in the Malay and Chinese datasets and adjusted for age, sex and population stratification (PC1-3 for Malay and PC1 for Chinese). All genome-wide SNPs were replicated, *in silico*, in the validation datasets. Subsequently, association summary statistics from discovery and validation stages were combined using the inverse variance-weighted meta-analysis, assuming a fixed effects model to derive overall association values. Cochran’s Q p-value (<0.05) and/or I^2^ (>40.0%) were used to evaluate for heterogeneity during meta-analysis. GWAS association analyses in the discovery and replication stages were also repeated using Genome-wide Efficient Mixed Model Association (GEMMA) that accounts for population stratification and sample structure during analysis^43^ and through the EIGENSTRAT method that corrects for stratification using continuous axes of variation^44^.

#### Association analysis with renal functions

Continuous variables with normal distribution were expressed as the mean ± standard deviation (SD), while non-normally distributed variables were presented as medians (25th-75th percentile). Categorical data were expressed as proportions. Comparison between ethnic groups or genotypes was performed by independent sample t-test for normally distributed variables or the Mann-Whitney *U* test or Kruskal-Wallis test for non-normally distributed variables (Table 1 and Supplementary Table S4). Comparisons of categorical variables were performed with chi-squared (χ2) test.

Binary logistic regression models were used to evaluate the association of SNP or HP allele (additive model) and DKD progression with the adjustment for age, gender, BMI, principle components, HbA1c, diabetes duration, systolic blood pressure, triglycerides, HDL-C, LDL-C, baseline eGFR and uACR and intake of RAS antagonist and insulin. Natural log transformed values for lipids and uACR was used for analysis (Table 3 and Supplementary Table S8). Association analysis of top SNP and ESRD were adjusted for age, sex, and PC components (PC1 for Chinese and PC1-3 for Malay, PCs not used for China samples without GWAS data) (Table 4). Statistical analyses were performed separately for the Chinese and Malay samples and a combined odd ratio was obtained using inverse-variance-weighted, fixed-effects meta-analysis (all P for heterogeneity > 0.05).

In the MR analysis, the causal estimate OR was derived using exp(β_2_/β_1_) as described by Burgess *et al*.^45^ where β_1_ = regression coefficient of IV and z transformed urine haptoglobin and β_2_ = regression coefficient of IV and DKD progression. The combined beta regression coefficients for association between instrumental variable (SNP and HP allele) and 1) uHP and 2) DKD progression was obtained from pool analysis of Chinese and Malay samples using the inverse variance-weighted meta-analysis, assuming a fixed effects model. A two-sided P values of <0.05 was considered statistically significant. All analysis were performed using SPSS version 22 and R software (version 3.3.2).

### Data availability

The datasets generated during and/or analyzed during the current study are available from the corresponding author on reasonable request.

## Electronic supplementary material

Supplementary Information
