# Author Correction: Genetic markers for urine haptoglobin is associated with decline in renal function in type 2 diabetes in East Asians

**DOI:** 10.1038/s41598-018-31990-6

**Published:** 2018-09-07

**Authors:** Resham Lal Gurung, Rajkumar Dorajoo, Sylvia Liu, Yiamunaa M, Jian-Jun Liu, Ling Wang, Lin Guo, Xueqing Yu, Jian-Jun Liu, Su Chi Lim

**Affiliations:** 1 Clinical Research Unit, Khoo Teck Puat Hospital, Singapore, Singapore; 2 Human Genetics, Genome Institute of Singapore, Agency for Science, Technology and Research, Singapore, Singapore; 3 The First Affiliated Hospital, Sun Yat-sen University, Guangzhou, China; 4 Guangdong Medical University, Guangzhou, China; 5 Yong Loo Lin School of Medicine, National University of Singapore, Singapore, Singapore; 6 Diabetes Centre, Khoo Teck Puat Hospital, Singapore, Singapore; 7 Saw Swee Hock School of Public Health, National University Hospital, Singapore, Singapore

Correction to: *Scientific Reports* 10.1038/s41598-018-23407-1, published online 23 March 2018

This Article contains typographical errors in the Acknowledgements section.

“We would like to thank all the research team members from Clinical Research Unit and clinical doctors from diabetes centre at Khoo Teck Puat Hospital, Singapore for assistance with participant recruitment and data input for the DN and SMART2D and follow-up study. This study was supported by Alexandra Health Enabling Grant (AHEG1504) and Small Innovation Grants (AHPL SIG I/13031 & STAR17109).”

should read:

“We would like to thank all the research team members from Clinical Research Unit and clinical doctors from diabetes centre at Khoo Teck Puat Hospital, Singapore for assistance with participant recruitment and data input for the DN and SMART2D and follow-up study. This study was supported by Alexandra Health Enabling Grant (AHEG1504), Small Innovation Grants (AHPL SIG I/13031 & STAR17109) and Singapore National Medical Research Council Grants (NMRC/PPG/AH (KTPH)/2011 and NMRC/CIRG/1398/2014).”

