# Supplementary material for: Genetic markers for urine haptoglobin is associated with decline in renal function in type 2 diabetes in East Asians
Source: Sci Rep. 2018 Mar 23;8:5109. doi: 10.1038/s41598-018-23407-1 (PMC5865208; doi:10.1038/s41598-018-23407-1)
Supplement: Supplementary file 1 — Supplementary Information [file 41598_2018_23407_MOESM1_ESM.pdf]

Supplementary Information

Genetic markers for urine haptoglobin is associated with decline in renal function in type 2 diabetes in East Asians

Resham Lal Gurung[1#], Rajkumar Dorajoo[2#], Sylvia Liu[1], Yiamunaa M[1], Jian-Jun Liu[1], Ling Wang[2], Lin Guo[3], Xueqing Yu[4], Jian-Jun Liu[2, 5 *], Su Chi Lim[1, 6, 7 *]

[1]Clinical Research Unit, Khoo Teck Puat Hospital, Singapore, [2] Human Genetics, Genome Institute of Singapore, Agency for Science, Technology and Research, Singapore, [3]The First Affiliated Hospital, Sun Yat-sen University, China, [4] Guangdong Medical University, China, [5]Yong Loo Lin School of Medicine, National University of Singapore, Singapore, [6]Diabetes Centre, Khoo Teck Puat Hospital, Singapore, [7]Saw Swee Hock School of Public Health, National University Hospital, Singapore

10 Supplementary tables

4 Supplementary figures

Supplementary Table S1. Details of DN cohort GWAS sample QC filters utilized in study.

Total samples genotyped	2,664
Samples with call-rate < 95%	4
Samples with exteremes in heterozygosity (> 3SD)	55
Known positive controls	12
Cryptic related samples	96
PCA outliers	127
Remaining samples	1,869 Chinese samples, 450 Malay samples and 51 Indian samples
Samples with phenotype	236 Chinese samples, 57 Malay samples and 45 Indian samples

Supplementary Table S2. Details of DN cohort GWAS SNP QC filters utilized in this study.

	**Chinese**	**Malays**
N	236	57
Total SNPs	853,994	853,994
Non-autosomal SNPs	23,250	23,250
SNPs with call-rate < 95%	523	945
Monomorphic and rare SNPs (MAF < 1%)	48,434	54,504
SNPs with HWE p-value < 10^{-4}	8	2
Remaining SNPs that pass QC	781,779	775,293

Supplementary Table S3: Comparison of association study results for novel SNP (rs75444904) and rs217181 in linear regression analysis adjusted for principle component 1 in Chinese datasets and principle components 1-3 in Malay dataset, linear regression adjusted for 10 eigenvalues in all datasets and GEMMA. All analyses were additionally adjusted for age and sex.

					DN Chinese (N=236)			DN Malay (N=57)			SMART2D Chinese (N=512)			Meta (N=805)			
**SNP**	**Chr**	**Pos**	**TA**	**OA**	**TAF**	**β (SE)**	**P**	**TAF**	**β (SE)**	**P**	**TAF**	**β (SE)**	**P**	**β (SE)**	**P**	**P_het**	**I^2**
Linear regression adjusted for principle component 1 in Chinese datasets and principle components 1-3 in Malay dataset																	
kgp16506790/ rs75444904	16	72095650	C	A	0.307	0.732 (0.082)	1.07E-16	0.219	0.705 (0.214)	0.0018	0.275	0.807 (0.059)	3.98E-41	0.777 (0.047)	1.21E-60	0.717	0
rs217181	16	72114002	T	C	0.335	0.592 (0.086)	5.33E-11	0.272	0.469 (0.226)	0.0316	0.319	0.648 (0.061)	2.11E-25	0.621 (0.048)	1.45E-36	0.667	10
Linear regression adjusted for 10 eigenvalues																	
kgp16506790/ rs75444904	16	72095650	C	A	0.307	0.710 (0.082)	5.65E-16	0.219	0.681 (0.215)	0.0040	0.275	0.800 (0.059)	2.32E-40	0.765 (0.047)	6.66E-59	0.621	0
rs217181	16	72114002	T	C	0.335	0.581 (0.086)	2.56E-11	0.272	0.438 (0.242)	0.0760	0.319	0.647 (0.061)	2.35E-25	0.617 (0.048)	1.44E-35	0.617	10
Mixed model regression analysis in GEMMA																	
kgp16506790/ rs75444904	16	72095650	C	A	0.307	0.727 (0.082)	2.22E-16	0.219	0.686 (0.214)	0.0024	0.275	0.810 (0.060)	5.04E-39	0.776 (0.047)	7.74E-60	0.653	0
rs217181	16	72114002	T	C	0.335	0.588 (0.086)	8.35E-11	0.272	0.451 (0.212)	0.0382	0.319	0.647 (0.061)	5.59E-24	0.619 (0.048)	3.88E-36	0.614	0

Abbreviations: Chr, chromosome number; TA, test allele; OA, other allele; TAF, test allele frequency; P_het, Cochran's Q heterogeneity p-value; I^2, heterogeneity index.

Supplementary Table S4. Urine and plasma haptoglobin levels among Haptoglobin genotype stratified by ethnicity.

	Chinese				Malay				Total		
	N	Progressors (n, %)	urine HP (ng/ml)	plasma HP (μg/ml)	N	Progressors (n, %)	urine HP (ng/ml)	plasma HP (μg/ml)	Progressors (n, %)[c,d]	urine HP (ng/ml)[a,b]	plasma HP (μg/ml)[a,b]
**HP genotype**											
HP 1-1	28	9	4667	1431	7	6	1659	1418	**15**	**4292**	**1421**
		(32.1)	(674-17048)	(1051-1835)		(85.7)	(1356-97227)	(1186-1697)	**(42.9)**	**(921-18050)**	**(1113-1754)**
HP 2-1	103	42	474	1277	27	17	1237	1554	**59**	**511**	**1327**
		(40.8)	(89-2213)	(952-1732)		(63.0)	(113-4883)	(930-1860)	**(45.4)**	**(91-2309)**	**(1005-1709)**
HP 2-2	119	32	28	867	43	17	159	982	**49**	**41**	**902**
		(26.9)	(2-195)	(473-1202)		(39.5)	(5-836)	(810-1287)	**(30.2)**	**(2-304)**	**(565-1158)**
**rs75444904**											
CC	28	9	4667	1419	1	1	10442	1527	**10**	**5041**	**1419**
		(32.1)	(433-17048)	(1007-1835)		(100)			**(34.5)**	**(457-16046)**	**(1014-1794)**
AC	89	38	474	1257	23	15	1657	1393	**53**	**584**	**1298**
		(42.7)	(96-2316)	(1008-1658)		(65.2)	(288-15271)	(1026-1680)	**(47.3)**	**(139-2532)**	**(1012-1671)**
AA	119	30	29	861	33	15	188	994	**45**	**54**	**895**
		(25.2)	(2-227)	(473-1126)		(45.5)	(7-743)	(822-1599)	**(29.6.)**	**(3-352)**	**(556-1192)**

Data described are median (interquartile range). [a] significant difference among HP genotype P<0.0001; [b]significant difference among rs75444904 P<0.0001

[c] significant difference among HP genotype P<0.05; [d]significant difference among rs75444904 P<0.05.

Supplementary Table S5. Conditional probability analysis of association of urine haptoglobin (zHCR) and HP genotype and rs75444904.

	**HP**				**HP adj for rs75444904**				**rs75444904**				**rs75444094 adj for HP**			
	**DN Chinese**	**DN Malay**	**SMART2D Chinese**	**Meta**	**DN Chinese**	**DN Malay**	**SMART2D Chinese**	**Meta**	**DN Chinese**	**DN Malay**	**SMART2D Chinese**	**Meta**	**DN Chinese**	**DN Malay**	**SMART2D Chinese**	**Meta**
N	236	57	500	793	236	57	500	793	236	57	512	805	236	57	500	793
β	0.790	0.628	0.708	0.727	0.616	0.516	0.074	0.264	0.732	0.705	0.807	0.777	0.186	0.193	0.740	0.543
SE	0.082	0.159	0.059	0.046	0.227	0.243	0.131	0.103	0.082	0.214	0.059	0.047	0.226	0.317	0.137	0.110
P	8.83E-19	7.50E-05	2.32E-32	**1.42E-55**	7.00E-03	0.039	0.572	**0.010**	1.07E-16	1.81E-03	**3.98E-41**	1.21E-60	0.410	0.545	7.05E-08	**8.22E-07**
P_het				0.580				0.069				0.717				0.056

Abbreviations: HP, Haptoglobin genotype; P_het, Cochran's Q heterogeneity p-value.

Supplementary Table S6. Genome-wide levels of associations for urine and plasma z-HP levels in the discovery stage of the study and their corresponding association levels in the validation datasets.

				Discovery stage			Replication stage						Meta-analysis (N=805)			
				DN Chinese(N=236)			DN Malay(N=57)			SMART2D Chinese(N=512)						
**SNP**	**Chr**	**Pos**	**TA**	**TAF**	**β (SE)**	**P**	**TAF**	**β (SE)**	**P**	**TAF**	**β (SE)**	**P**	**β (SE)**	**P**	**P_het**	**I2**
urine z-HP																
rs75444904	16	72095650	C	0.307	0.732 (0.082)	1.07E-16	0.219	0.705 (0.214)	1.80E-03	0.275	0.807 (0.059)	3.98E-41	0.777 (0.047)	1.21E-60	0.717	0
rs217181	16	72114002	T	0.335	0.592 (0.086)	5.33E-11	0.272	0.469 (0.212)	3.20E-02		0.648 (0.061)	2.11E-25	0.621 (0.048)	1.45E-36	0.667	0
Plasma z-HP																
rs75444904	16	72095650	C	0.307	0.583 (0.088)	2.08E-10	0.219	0.438 (0.251)	0.087	NA	NA	NA	0.567 (0.083)	6.51E-12	0.586	0
rs217181	16	72114002	T	0.335	0.414 (0.091)	9.38E-06		0.254 (0.238)	2.93E-01	NA	NA	NA	0.384 (0.084)	4.24E-06	0.329	0.7

Data analysis was adjusted for age, sex and Principal Components (PC1 for Chinese and PC1-3 for Malay). **Abbreviations**: Chr, chromosome number; TA, test allele; TAF, test allele frequency; P_het, Cochran's Q heterogeneity p-value;I^2, heterogeneity index; NA, not available.

Supplementary Table S7. Conditional probability analysis of association of urine haptoglobin (zHCR) and rs217181 and rs75444904.

	**rs217181**				**rs217181 adj for rs75444904**				**rs75444904**				**rs75444094 adj for rs217181**			
	**DN Chinese**	**DN Malay**	**SMART2D Chinese**	**Meta**	**DN Chinese**	**DN Malay**	**SMART2D Chinese**	**Meta**	**DN Chinese**	**DN Malay**	**SMART2D Chinese**	**Meta**	**DN Chinese**	**DN Malay**	**SMART2D Chinese**	**Meta**
N	236	57	512	805	236	57	512	805	236	57	512	805	236	57	512	805
β	0.592	0.469	0.648	0.621	-0.418	-0.298	-0.262	-0.308	0.732	0.705	0.80667	0.777	1.11	0.967	1.048	1.060
SE	0.086	0.212	0.061	0.048	0.195	0.372	0.128	0.103	0.082	0.214	0.059	0.047	0.196	0.397	0.131	0.105
P	5.33E-11	3.20E-02	2.11E-25	**1.45E-36**	0.033	0.428	0.040	**0.003**	1.07E-16	1.80E-03	3.98E-41	**1.21E-60**	1.60E-08	0.018	1.55E-15	**8.64E-23**
P_het				0.660				0.799				0.717				0.938

Data analysis was adjusted for age, sex and Principal Components (PC1 for Chinese and PC1-3 for Malay). **Abbreviations**: P_het, Cochran's Q heterogeneity p-value.

Supplementary Table S8. Association of rs75444904 and HP 1 allele and DKD progression.

	HP1			rs75444904		
	**DN Chinese**	**DN Malay**	**Meta**	**DN Chinese**	**DN Malay**	**Meta**
OR	1.66	6.13	1.91	1.66	3.03	1.77
95% CI	1.17-2.14	4.73-7.53	1.45-2.37	1.18-2.14	1.64-4.43	1.32-2.23
P	0.042	0.011	**0.006**	0.038	0.119	**0.014**
P_het			0.084	0.799		0.425

Data represents odd ratios (OR) and 95% CI adjusted for age, sex, BMI, HbA1c, diabetes duration, systolic BP, TG, LDL-C, HDL-C, eGFR, uACR and RAS antagonist and insulin usage. Abbreviations: P_het, Cochran's Q heterogeneity p-value. Bold values represent statistically significant data.

Supplementary Table S9. Number of participants (n, %) in each rs75444904 genotype in the validation study cohorts.

rs75444904 Genotype	DN Chinese			DN Malay			SMART2D Chinese			SMART2D Malay			Chinese		
	Case	Control	Total	Case	Control	Total	Case	Control	Total	Case	Control	Total	Case	Control	Total
AA	91 (20.6)	350 (79.4)	441 (50.1)	56 (42.4)	76 (57.6)	132 (58.9)	7 (3.3)	203 (96.7)	210 (53.7)	7 (11.9)	52 (88.1)	59 (56.2)	38 (64.4)	21 (35.6)	59 (50.4)
CA	81 (23.4)	265 (76.6)	346 (39.3)	37 (47.4)	41 (52.6)	78 (34.8)	10 (6.5)	143 (93.5)	153 (39.1)	5 (16.7)	25 (83.3)	30 (28.6)	33 (78.6)	9 (21.4)	42 (35.9)
CC	22 (23.4)	72 (76.6)	94 (10.7)	9 (64.3)	5 (35.7)	14 (6.3)	1 (3.6)	27 (96.4)	28 (7.2)	2 (12.5)	14 (87.5)	16 (15.2)	11 (68.8)	5 (31.3)	16 (13.7)

Frequency of genotype within ESRD (Case) or Control group in independent validation cohort.

Supplementary Table S10. Mediation analysis of urine haptoglobin or LDL cholesterol on the HP1 and DKD progression adjudged for traditional DKD risk factors age, gender, ethnicity, HbA1c, Diabetes duration, SBP, TG, eGFR and uACR.

X=HP1 , M=uHP, Y= DKD progression	β	P
X--> Y	**0.600**	**0.005**
X--> M	**2.502**	**<0.0001**
M-->Y	**0.183**	**<0.0001**
X+ M--> Y		
X	0.242	0.320
M	**0.158**	**0.003**
X + M + X*M--> Y		
X	0.496	0.356
M	**0.177**	**0.006**
X*M	-0.038	0.595

X=HP1 , M=LDL-C, Y= DKD progression	β	P
X--> Y	**0.600**	**0.005**
X--> M	0.040	0.069
M-->Y	0.905	0.081
X+ M--> Y		
X	**0.577**	**0.007**
M	0.801	0.130

Regression coefficient (β) and P values are given. Bold values represent statistically significant data.

Supplementary Figure S1. PCA plot (PC1 vs PC2) of DN dataset Chinese, Malay and Indian subjects with 1000 Genome reference populations. Samples in yellow boxes identified as outliers and excluded from analyses.

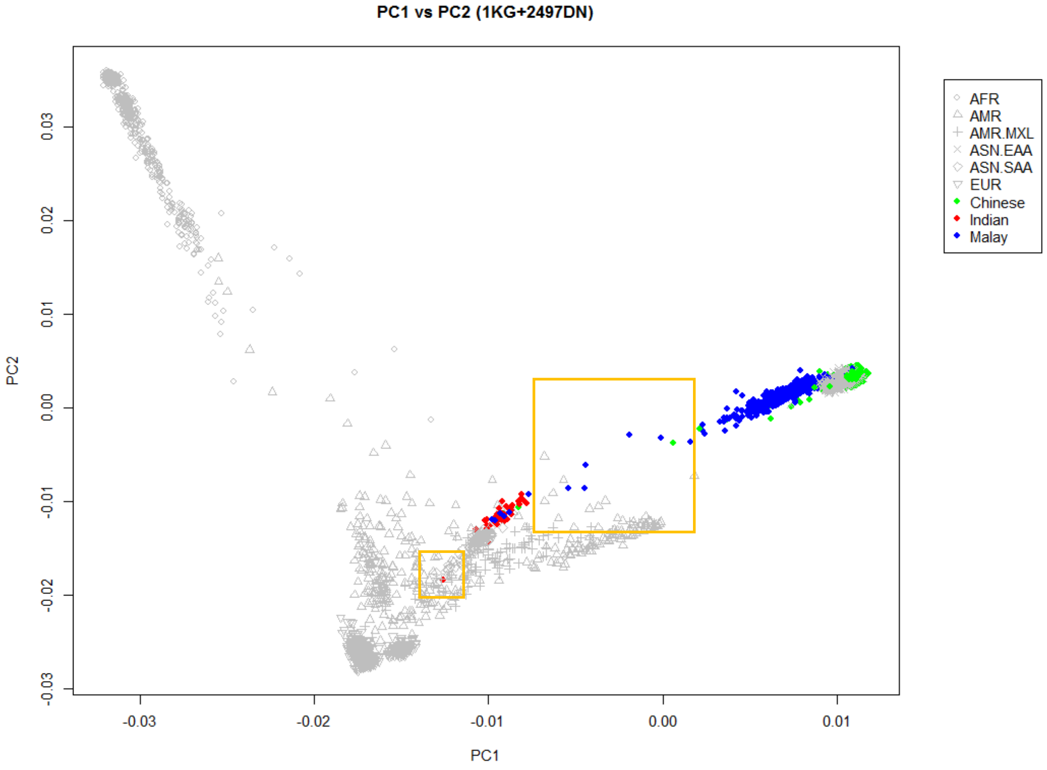

Supplementary Figure S2. PCA plot (PC1 vs PC2) of DN dataset Chinese, Malay and Indian subjects. Samples in yellow boxes assigned as Indian, Malay and Chinese ethnic groups.

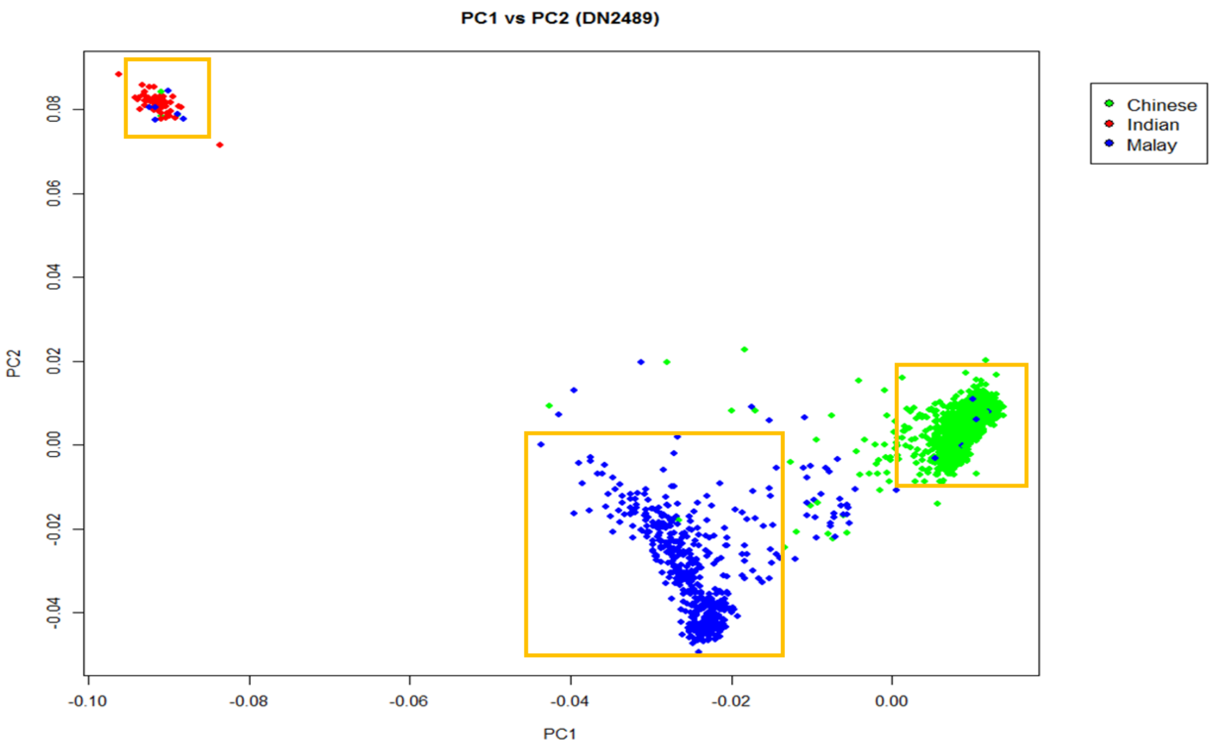

Supplementary Figure S3. QQ-plot of observed and expected p-values for urine z-HP associations in A) discovery stage DN Chinese samples (N=236, λ = 1.003); B) validation stage DN Malay samples (N=57, λ = 1.027); C) validation stage SMART2D Chinese samples (N=512, λ = 1.004).

A

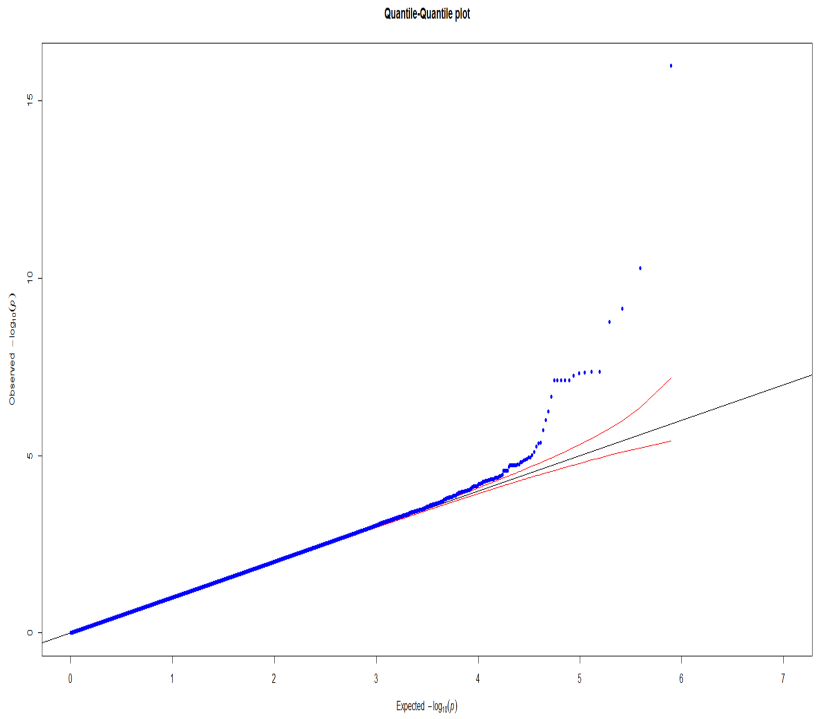

B

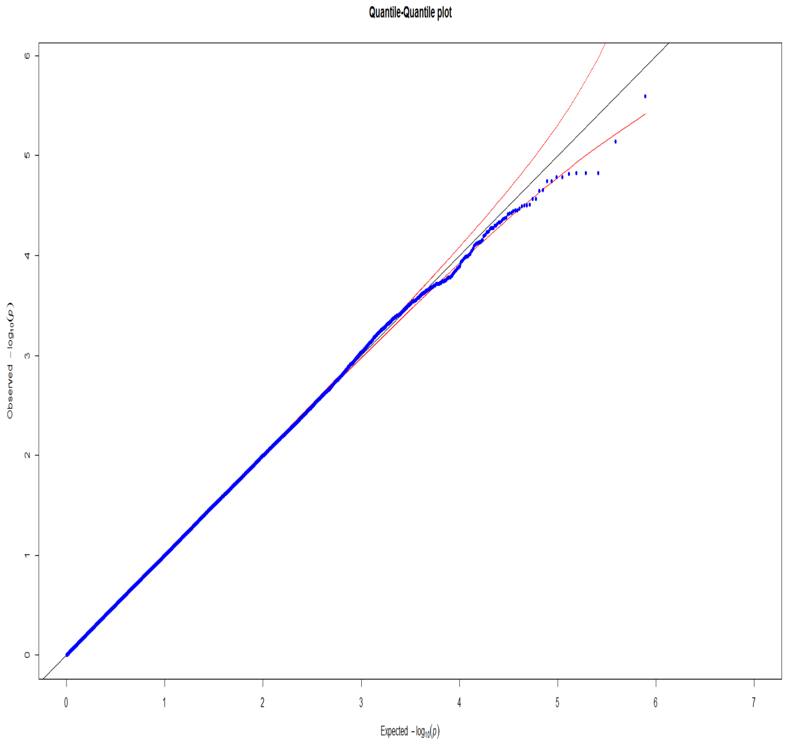

C

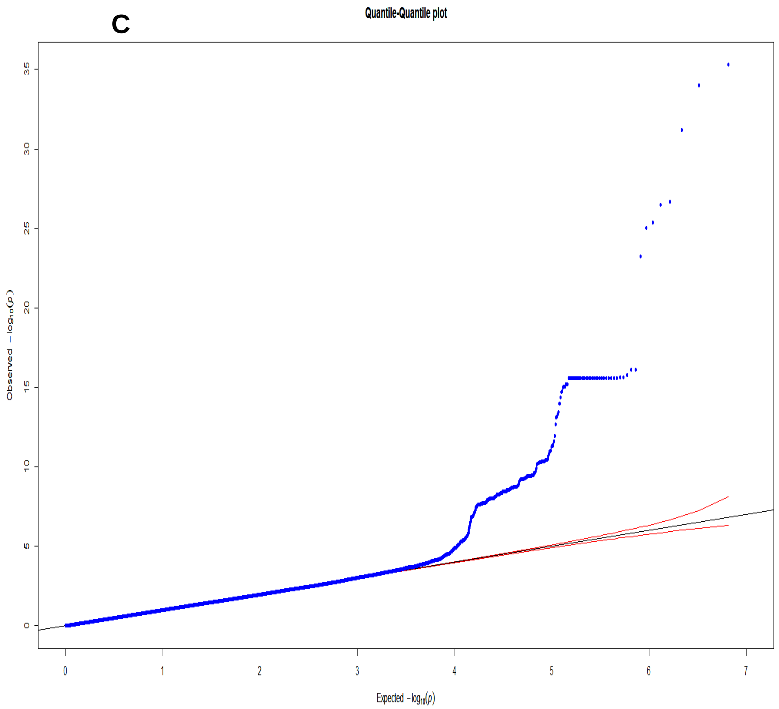
Quantile-Quantile plot
Observed −log10(p)
Expected −log10(p)
0
5
10
15
20
25
30
35
0
1
2
3
4
5
6
7

Supplementary Figure S4. QQ-plot of observed and expected p-values for plasma z-HP associations in A) discovery stage DN Chinese samples (N=236, λ = 0.999) and B) validation stage DN Malay samples (N=57, λ = 0.990).

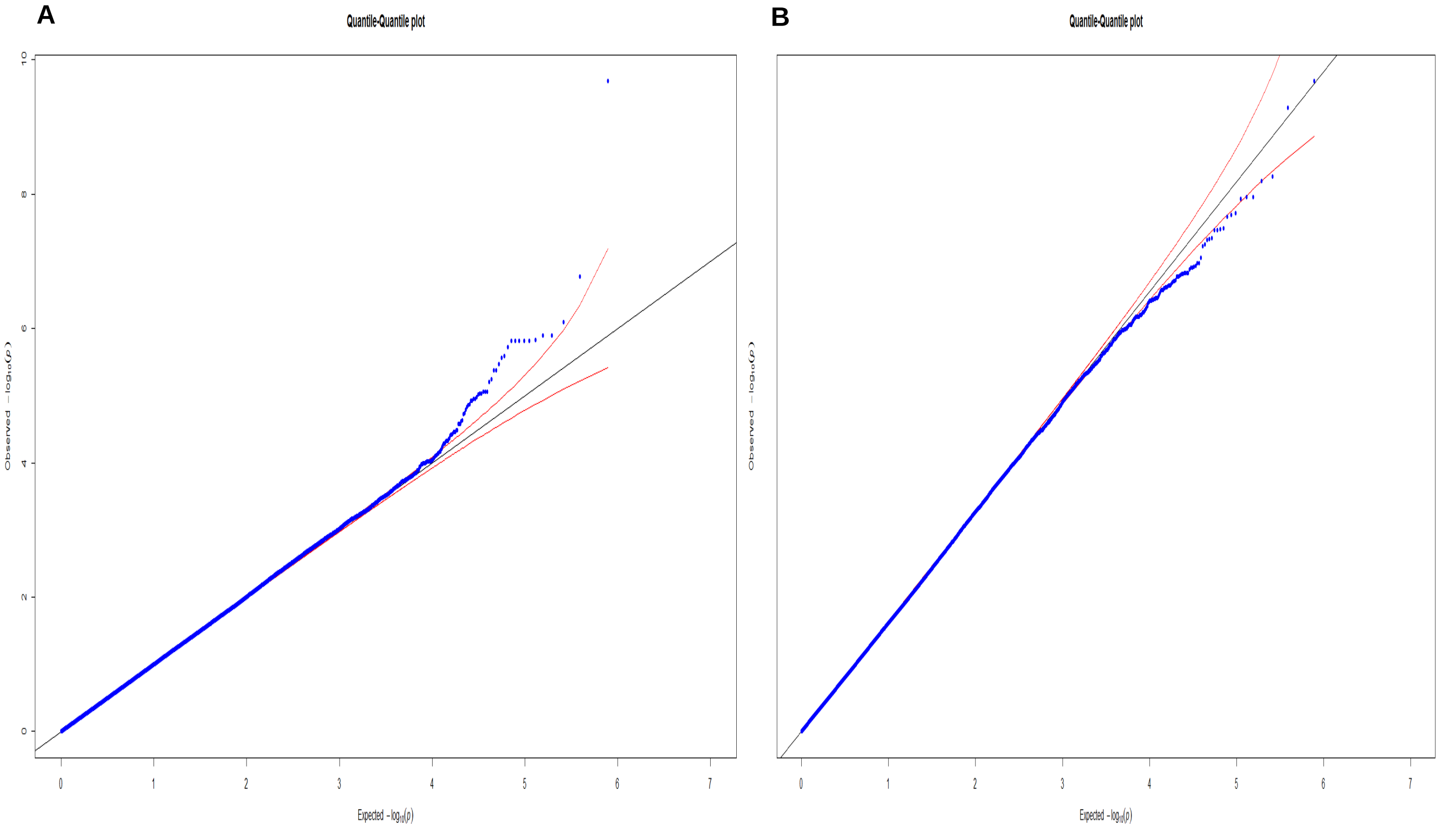

Supplementary Methods:

Participant's recruitment details in Chinese (Ch) validation Cohort:

The Chinese samples consist of 82 T2D-related ESRD cases and 34 T2D-related controls and were collected during the period from October 2015 to December 2016. The ESRD cases samples were collected from 7 hospitals, The First Affiliated Hospitals of Sun Yat-sen University, The Third Affiliated Hospitals of Sun Yat-sen University, Kaiping Central Hospital, Guizhou Provincial People's Hospital, Jieyang People's Hospital, The First Affiliated Hospital of Zhengzhou University and Lanzhou University Second Hospital. Participants who had been diagnosed type 2 diabetic kidney disease with an eGFR less than 15mL/min/1.73m^2 were recruited in cases group. The T2D-related controls samples were collected from the First Affiliated Hospitals of Sun Yat-sen University. Patients who had been diagnosed type 2 diabetes for more than 10 years without proteinuria or microvascular were recruited. Written informed consent forms were obtained from all participants.
